# The Mechanisms of Plasticity of Nociceptive Ion Channels in Painful Diabetic Neuropathy

**DOI:** 10.3389/fpain.2022.869735

**Published:** 2022-03-28

**Authors:** Sonja L. Joksimovic, Vesna Jevtovic-Todorovic, Slobodan M. Todorovic

**Affiliations:** ^1^Department of Anesthesiology, University of Colorado Denver, Aurora, CO, United States; ^2^Neuroscience Graduate Program, University of Colorado Denver, Aurora, CO, United States

**Keywords:** T-type calcium channel 3.2, diabetic neuropathy, mechanisms of pain, ion channels, plasticity

## Abstract

Treating pain in patients suffering from small fiber neuropathies still represents a therapeutic challenge for health care providers and drug developers worldwide. Unfortunately, none of the currently available treatments can completely reverse symptoms of either gain or loss of peripheral nerve sensation. Therefore, there is a clear need for novel mechanism-based therapies for peripheral diabetic neuropathy (PDN) that would improve treatment of this serious condition. In this review, we summarize the current knowledge on the mechanisms and causes of peripheral sensory neurons damage in diabetes. In particular, we focused on the subsets of voltage-gated sodium channels, TRP family of ion channels and a Ca_V_3.2 isoform of T-type voltage-gated calcium channels. However, even though their potential is well-validated in multiple rodent models of painful PDN, clinical trials with specific pharmacological blockers of these channels have failed to exhibit therapeutic efficacy. We argue that understanding the development of diabetes and causal relationship between hyperglycemia, glycosylation, and other post-translational modifications may lead to the development of novel therapeutics that would efficiently alleviate painful PDN by targeting disease-specific mechanisms rather than individual nociceptive ion channels.

## Introduction

According to the International Diabetes Federation, more than 400 million people suffer from diabetes worldwide, and by 2045 this number will increase to 700 million (1). This places diabetes on the list of the great epidemics of the 21st century. In the US alone, more than 30 million people are diagnosed and treated for diabetes, while at the same time more than 90 million have been diagnosed with prediabetic condition (2).

Diabetes mellitus is a chronic condition accompanied by numerous complications, with neuropathies and diabetic foot (due to hyperglycemia induced damage to foot nerves and blood vessels) occurring in more than 50% of the patients (3). Neuropathic pain represents a type of a painful state commonly caused by lesions or chronic disease affecting the somatosensory nervous system. The most prevalent neuropathic complication of diabetes is distal symmetric polyneuropathy (peripheral diabetic neuropathy) manifested as a loss of distal sensory function of the lower extremities. Patients exhibiting peripheral diabetic neuropathy (PDN) often experience increased incidence of falling due to affected proprioception and painful sensations such as tingling, burning sensation on the feet (spontaneous stimulus-independent pain), or hyperalgesia to heat and touch (evoked, stimulus-dependent pain). Some patients might even experience paroxysmal sharp deep pain, while in some patients over time the painful sensations subside and become replaced by the absence of pain and sensations perception. In some patients, an increased sensitivity to a typically non-painful stimulus can also occur (brush allodynia). This diversity of different symptoms of altered pain perception are a good indicator of the complexity of the PDN and challenges for its treatment. Finally, regardless of the symptoms, either presence of painful sensitivity or pathological loss of peripheral nerve sensitivity represent serious sequalae leading to a reduced quality of life.

The treatment options clinically used to alleviate the symptoms of painful PDN are very limited in their efficacy and often accompanied with numerous side effects. Most used therapeutics in the clinical setting are gabapentionoids, such as pregabalin and gabapentin, both considered to reduce pain by targeting regulatory subunit α2δ of Ca_V_2.2 (N-type) isoform of voltage-gated calcium channels. However, the use of these drugs is not devoid of side effects, and more than half of patients treated with gabapentinoids experience various side effects such as sedation, ataxia and dizziness as well as weight gain (4). Furthermore, recent reports indicate that misuse and drug abuse of gabapentinoids is on the rise (5, 6).

## The Mechanisms of the Development of Peripheral Diabetic Neuropathy

Peripheral diabetic neuropathy is a neurodegenerative disorder of the peripheral nervous system affecting predominantly sensory as well as autonomic nerves, and to a lesser extent motor nerves (7). Patients suffering from diabetes type II are twice as likely to experience symptoms of painful PDN than patients with diabetes type I (4). The exact mechanisms of the onset of PDN are still not clear, and factors such as hyperglycemia, hyperlipidemia, microvascular changes of the blood supply to peripheral nerves as well as impaired insulin signaling could contribute to peripheral nerve damage. Development of type I diabetes in rodents leads to the changes in the expression of neurofilament thus affecting the integrity of peripheral axons and neuronal somas (8) within the dorsal root ganglia (DRG), which could in turn induce changes in peripheral sensory painful neuronal transmission. Also, chronic hyperglycemia contributes to the Schwan cell damage, which could in turn lead to demyelination (9, 10) and axonal alterations, such as dysregulation of cytoskeletal properties (11) and axonal transport (12). Both *in vivo* and *in vitro* rodent models have demonstrated that hyperglycemia leads to the functional alterations in numerous proteins expressed in the DRGs [such as neuromodulin, β-tubulin, as well as heat-shock proteins and poly(ADP-ribose) polymerase PARP] that play an important role in protein processing (13), oxidative stress and mitochondrial function (14, 15), which could affect central pain processing leading to peripheral nerve dysfunction. Furthermore, enzymatic post-translational modification of various proteins *via* glycosylation is known to regulate protein conformation and stability in cell membranes, trafficking and secretion thus affecting its functional properties (16). Of particular importance is asparagine (N)-linked glycosylation involving extracellular asparagine residues. It was previously shown that in embryonic DRG neurons, neuraminidase (NEU), an enzyme that deglycosylates proteins by removing sialic acid residues, affected steady-state inactivation of voltage-gated sodium channels (17). The glycosylation of transient receptor potential (TRP) channels such as TRPV1 and TRPM8 is also known to modulate their function, thus potentially altering the cellular excitability of DRG sensory neurons (18, 19).

### The Ion Channels and Their Role in Neuronal Hyperexcitability in Painful PDN

The specific and crucial peripheral positioning of the primary sensory afferents as distal sites of the generation of action potential has led to the extensive research of the underlying mechanisms of neuronal hyperexcitability and changes in baseline thresholds in various pain disorders. The key contributors to the neuronal transduction and transmission are nociceptive ion channels, and the alterations in their expression and trafficking, or their functional changes driven by phosphorylation or glycosylation can significantly affect the neuronal excitability and contribute to the pathophysiology of neuropathic painful states.

As part of the pursuit for novel analgesics, voltage-gated sodium channels have been extensively investigated for their important role in pathophysiology of pain. Among nine different Na_V_ channel subtypes, Na_V_1.7, Na_V_1.8 as well as Na_V_1.9 have been recognized as essential for excitability of sensory neurons, especially since their expression is enriched in nociceptors. Among them, Nav1.7 and Nav1.9 are important for determining the action potential threshold, thus being responsible for the excitability of the primary afferent neurons and sensory signal amplification, whilst Nav1.8 channels are essential for the upstroke of the action potential in nociceptors (20).

One of the pivotal findings was the discovery that patients with inherent inability to sense pain possess non-functional Nav1.7 channels expressed in the DRGs due to the existence of different mutations of the SCN9A gene encoding these channels (21). Therefore, the inability of primary sensory neurons to generate and maintain action potentials leads to the absence of pain sensitivity. On the other hand, gain-of- function variants in SCN9A (encoding Nav1.7) are present in a few pain disorders, such as erythromelalgia and small-fiber neuropathy (21).

Primary sensory neurons in painful PDN can become hyperexcitable and spontaneously active, leading to the central sensitization and pathological activation of the pain pathways. Rare variants in SCN9A (Na_V_1.7 channels) were discovered in patients with painful PDN, however whether these variants are specific for this condition only is yet to be understood (22). A clinical trial with a small-molecule selective Nav1.7 blocker developed as a treatment for painful PDN yielded negative results (23). Perhaps, investigating further the contribution of SCN9A variants in PDN would provide better understanding of the role of these channels in the development of painful PDN, and appropriate approach of developing novel Na_V_1.7 inhibitors efficient for treating painful PDN.

In rodent models of painful PDN, reported increase of the expression of Na_V_1.8 channels in C fibers facilitated impulse conduction and central sensitization, collectively creating the environment for the neuropathic pain development (24). Furthermore, methylglyoxal, reactive metabolite enriched in diabetes (25), post-translationally modifies Nav1.8 channels, resulting in Nav1.8 gain of function that facilitates increased sensory neuronal firing, contributing to hyperalgesia in rodent models of diabetic neuropathy. The effects of methylglyoxal are not selective only for sodium channels. Transient receptor potential (TRP) channels, involved in signal transduction, can also be affected by methylglyoxal. Andersson et al. (26) have recently shown that methylglyoxal represents a potent intracellular TRPA1 channel agonist, and locally applied exerts pain like behavior in rodents. Furthermore, local injections of methylglyoxal induced pain in humans through C-fiber sensitization mostly by activating TRPA1 channels (27). TRPA1 channels are present in the peripheral nerve terminals, central synapse and on non-neuronal cells [for detailed review refer to (28)] therefore pharmacological or genetic attenuation of TRPA1 signaling reduced mechanical hyperalgesia (29) and cutaneous blood flow (30, 31). Intrathecal application of TRPA1 antagonist required lower dose of the compound to achieve antihyperalgesic effect than systemic or intraplantar application (29). TRPA1 antagonist GRC 17356 developed by Glenmark Pharmaceuticals S.A. has been investigated in phase II clinical trial in patients with diabetic peripheral neuropathy, however, thus far the results of this trial are not known (ClinicalTrials.gov).

Similarly, the dysregulation of capsaicin-gated TRPV1 channels, a typical representative of the vanilloid family of ligand-gated channels, contributes to pathological sensory signaling and pain in rodent models of diabetes (32). Previous studies have discovered an increase of the membrane expression of TRPV1 channels in myelinated A fibers only (33), while Pabbidi et al. (34) detected increased both expression of TRPV1 and whole-cell currents in small DRG cells in both STZ-induced and transgene-mediated type 1 painful PDN in mice. Facer et al. (35), however, discovered a reduction of TRPV1 channels, possibly due to overall loss of TRPV1 rich fibers and general downregulation of TRPV1 in diabetic patients.

### The Role of T-Channels in Pain

Low voltage-activated calcium channels (LVA or T-Type Ca2+ channels) have been previously investigated in neuronal excitability and nociceptive transmission. They were first discovered in acutely dissociated sensory neurons (36) of small size (cell soma <31 μm). The channel's unique biophysical characteristics, such as activation by small membrane depolarization, relatively fast, and voltage-dependent inactivation kinetics, as well as slow deactivation kinetics upon channel closing following maximal activation, assigns a very important role in controlling cellular excitability of sensory neurons. These properties have been confirmed in a subpopulation of acutely dissociated rat DRG cells of medium size (cell soma 32–45 μm) where T-channels controlled sub-threshold excitability of DRG cells by lowering the threshold for action potential generation (37).

Nelson et al. (38) showed the majority of capsaicin-sensitive (TRPV1 rich) small DRG neurons typically considered nociceptive neurons also express T-currents. Particularly, a subtype of T-channels, Ca_V_3.2, has been critical for regulating cellular excitability of capsaicin-sensitive, TTX-r Na_V_1.8 and Na_V_1.9 and isolectin B4 (IB4) positive nociceptive DRG neurons termed “T-rich” cells (39).

T-channels are also present in a subpopulation of DRG sensory neurons of medium size, involved in either nociception (IB4 positive) (40), or touch sensation (D-hair mechanoreceptors) (41). In addition, it was recently discovered that Cav3.2 channels play a crucial role in touch/pain pathophysiology, since they are present in the cutaneous low-threshold mechanoreceptors (LTMR) Aδ and C, both in the initial segment of the axon and in the peripheral terminals, as well as in the nodes of Ranvier of the LTMR-Aδ (42).

At the spinal level, T-channels were first discovered postsynaptically on the somas of the neurons of the dorsal horn superficial laminae, possibly regulating the activity-dependent synaptic strength between pre- and post-synaptic neurons (43, 44). Furthermore, Jacus et al. (45) discovered the presynaptic presence of Ca_V_3.2 channels in the central terminals of both peptidergic and nonpeptidergic fibers, suggesting the additional regulatory role of T-channels in controlling glutamate-mediated excitatory spontaneous transmission in nociceptive neurons in the central synapse. Similarly, they are found pre- and post-synaptically in dorsal horn laminae II–III of the spinal cord (42).

Several studies have shown that the expression of Ca_V_3.2 channels is increased in spinal dorsal horn or DRG neurons in models of chronic nerve injury (46–51), inflammatory pain models (52–56), acute pain model (57) as well as in chemically induced neuropathies (58) including diabetic neuropathy (40, 59).

## The Role of T-Channels in Painful PDN

Latham et al. (60), who used genetically altered animals lacking the functional Ca_V_3.2 T-channel globally, discovered that these animals upon streptozotocin (STZ) treatment, became hyperglycemic but did not develop diabetic neuropathy, as compared to their littermates. These data strongly suggest that the Ca_V_3.2 isoform of T-channels is necessary for development of STZ-induced painful PDN. Messinger et al. (59) discovered that in a STZ-induced diabetes type I in rats, insulin treatment lead to reversal of upregulation of T-currents in DRG neurons. Furthermore, diabetic animals treated with insulin exhibited reduction of pain-like behavior (reversal of thermal and mechanical hypersensitivity). Selective *in vivo* knock-down of the Ca_V_3.2 isoform of T-channels in DRG neurons reduced both hyperalgesia *in vivo*, and T-currents in dissociated DRG neurons of small diameter *in vitro*, suggesting a correlation between hyperglycemia and increased T-currents with the appearance of mechanical and thermal sensitivity in diabetic STZ-treated rats (59, 61). *In vivo* application of glycosylation inhibitors, such as NEU both peripherally and centrally, ameliorated hyperalgesia in a model of type I diabetes in rodents, except in Ca_V_3.2 knock-out mice (62). Similarly, recent study by (63) revealed that NEU exhibits desensitizing effects in terms of spontaneous activity and stimulated release of CGRP (calcitonin gene related peptide). Furthermore, the authors show that stimulated CGRP release in the sciatic nerve is markedly reduced with selective T-channel blocker TTA-P2 in healthy but not in diabetic wildtype and Ca_V_3.2 KO mice, suggesting that diabetes abolishes Ca_V_3.2-mediated activity in peripheral sensory nerves. TTA-P2 reduced KCl stimulated CGRP release from the hairy skin of wildtype but not Ca_V_3.2 knock-out mice, suggesting a *de novo* expression or re-distribution of these channels of cutaneous nerve endings of peptidergic nociceptive fibers. It is reasonable to speculate that this shift of T-channel expression in diabetic animals could in turn facilitate spontaneous neuronal activity.

In animal model of type 2 diabetes leptin-deficient (ob/ob) mice, mechanical and thermal hyperalgesia coincided with hyperglycemia observed early in life of these animals (60), and was reversible with insulin pretreatment. Additionally, the biophysical and biochemical alterations of predominantly Ca_V_3.2 subtype, such as T-current increase and T-channel mRNA expression, accompanied the development of painful PDN and hyperglycemia, indicating that metabolic changes leading to hyperglycemia are affecting DRG T-current in a similar causative fashion in both type I and II painful PDN, leading to the hyperexcitability of peripheral sensory neurons underlying hyperalgesia and allodynia.

Orestes et al. (64) discovered that NEU inhibited native T-currents in DRG neurons *in vitro*, and alleviated mechanical hyperalgesia *in vivo* in leptin-deficient (ob/ob) morbidly obese mouse model of type 2 painful PDN. This crucial impact of the channel glycosylation on its functionality was confirmed by Weiss et al. (65) that discovered that N-asparagine glycosylation at asparagine N192 is critical for channel surface expression, whereas glycosylation at asparagine N1466 controls the activity of Ca_V_3.2 T-channels. Additionally, noncanonical sites have also been recently discovered, namely asparagine N345 and N1780 located in the non-canonical N-glycosylation motifs N-X-C (NVC and NPC, respectively, with C being cysteine) essential for the expression of the human Cav3.2 channel in the plasma membrane (66).

These studies have firmly established that the Ca_V_3.2 isoform of T-channels in DRG cells is required for the development and maintenance of painful PDN in rats and mouse models of type 1 diabetes.

### Dysregulation of the Neuronal Transmission in the Spinal Cord—Potential Role of T-Channels

The upregulation of Ca_V_3.2 channels has been discovered within DRG neurons in various rodent models of chronic pain (48, 52, 58, 67, 68), therefore, they can be considered as a novel target to treat pain peripherally. However, ABT-639, a peripherally restricted T-channel antagonist, failed to alleviate pain in both clinical trials (37, 69) and rodent studies (70), raising the possibility that we might need centrally acting T-channel antagonists to alleviate pain effectively.

Although T-channel antagonists applied intrathecally reversed pain hypersensitivity in various rodent models of pain (49, 57, 62, 70), the presence of T-type mediated calcium currents in a subset of lamina II neurons was discovered only recently (71, 72). Harding et al. (73) discovered that T-type channels are available abundantly in lamina I neurons, and are dominant mediators of action potential-evoked calcium signals that represent actively backpropagating action potentials (73), shaping the excitability of neurons and affecting short-term and long-term plasticity in the spinal cord. Furthermore, Z944, a selective centrally penetrant T-channel antagonist, reduced superficial dorsal horn excitability *in vitro*, and reversed tactile allodynia in an inflammatory pain model in rats of both sexes. Perhaps future studies are needed to further elucidate the role and mechanisms of plasticity of centrally located T-channels in the development and maintenance of painful PDN.

## Discussion

Several ligand-gated and voltage-gated ion channel targets have great potential for the development of novel therapeutics to alleviate painful PDN. Here, we focused on subsets of voltage-gated sodium channels, TRP family of ion channels and Ca_V_3.2 isoform of T-type voltage-gated calcium channels. Although their potential is well-validated in multiple rodent models of PDN, clinical trials with specific pharmacological blockers of these channels have failed to exhibit therapeutic efficacy. Perhaps understanding the development of diabetes and causal relationship between hyperglycemia, glycosylation, and other post-translational modifications may lead to the development of novel therapeutics that would efficiently alleviate painful PDN by targeting disease-specific mechanisms rather than individual nociceptive ion channels.

## Author Contributions

SJ and ST conceptualized the manuscript. SJ, VJ-T, and ST wrote the manuscript. All authors contributed to the article and approved the submitted version.

## Funding

This study was supported by NIH grant NS091353 to ST and VJ-T.

## Conflict of Interest

The authors declare that the research was conducted in the absence of any commercial or financial relationships that could be construed as a potential conflict of interest.

## Publisher's Note

All claims expressed in this article are solely those of the authors and do not necessarily represent those of their affiliated organizations, or those of the publisher, the editors and the reviewers. Any product that may be evaluated in this article, or claim that may be made by its manufacturer, is not guaranteed or endorsed by the publisher.
